# Multi‐modality machine learning approach for risk stratification in heart failure with left ventricular ejection fraction ≤ 45%

**DOI:** 10.1002/ehf2.12929

**Published:** 2020-10-23

**Authors:** Gary Tse, Jiandong Zhou, Samuel Won Dong Woo, Ching Ho Ko, Rachel Wing Chuen Lai, Tong Liu, Yingzhi Liu, Keith Sai Kit Leung, Andrew Li, Sharen Lee, Ka Hou Christien Li, Ishan Lakhani, Qingpeng Zhang

**Affiliations:** ^1^ Xiamen Cardiovascular Hospital Xiamen University Xiamen China; ^2^ Tianjin Key Laboratory of Ionic‐Molecular Function of Cardiovascular Disease, Department of Cardiology, Tianjin Institute of Cardiology Second Hospital of Tianjin Medical University Tianjin 300211 China; ^3^ Faculty of Health and Medical Sciences University of Surrey GU2 7AL Guildford UK; ^4^ School of Data Science City University of Hong Kong Hong Kong SAR China; ^5^ Laboratory of Cardiovascular Physiology Li Ka Shing Institute of Health Sciences Hong Kong China; ^6^ Department of Anaesthesia and Intensive Care, Faculty of Medicine Chinese University of Hong Kong Hong Kong SAR China; ^7^ Aston Medical School Aston University Birmingham UK; ^8^ Faculty of Science University of Calgary Calgary AB Canada; ^9^ Faculty of Medicine Newcastle University Newcastle UK

**Keywords:** Heart failure, P‐wave, Inter‐atrial block, Strain, Neutrophil, Prognostic nutritional index

## Abstract

**Aims:**

Heart failure (HF) involves complex remodelling leading to electrical and mechanical dysfunction. We hypothesized that machine learning approaches incorporating data obtained from different investigative modalities including atrial and ventricular measurements from electrocardiography and echocardiography, blood inflammatory marker [neutrophil‐to‐lymphocyte ratio (NLR)], and prognostic nutritional index (PNI) will improve risk stratification for adverse outcomes in HF compared to logistic regression.

**Methods and results:**

Consecutive Chinese patients referred to our centre for transthoracic echocardiography and subsequently diagnosed with HF, between 1 January 2010 and 31 December 2016, were included in this study. Two machine learning techniques, multilayer perceptron and multi‐task learning, were compared with logistic regression for their ability to predict incident atrial fibrillation (AF), transient ischaemic attack (TIA)/stroke, and all‐cause mortality. This study included 312 HF patients [mean age: 64 (55–73) years, 75% male]. There were 76 cases of new‐onset AF, 62 cases of incident TIA/stroke, and 117 deaths during follow‐up. Univariate analysis revealed that age, left atrial reservoir strain (LARS) and contractile strain (LACS) were significant predictors of new‐onset AF. Age and smoking predicted incident stroke. Age, hypertension, type 2 diabetes mellitus, chronic kidney disease, mitral or aortic regurgitation, P‐wave terminal force in V1, the presence of partial inter‐atrial block, left atrial diameter, ejection fraction, global longitudinal strain, serum creatinine and albumin, high NLR, low PNI, and LARS and LACS predicted all‐cause mortality. Machine learning techniques achieved better prediction performance than logistic regression.

**Conclusions:**

Multi‐modality assessment is important for risk stratification in HF. A machine learning approach provides additional value for improving outcome prediction.

## Introduction

Heart failure (HF) is a growing epidemic and is estimated to affect around 26 million globally.^1^ It can lead to significant morbidity and mortality and is associated with significant health care expenditures through repeated hospitalizations, as well as hospital length of stay.^2^ Its underlying pathophysiological mechanisms are complex and are dependent on an interplay between increased oxidative stress, activation of pro‐inflammatory pathways, neurohumoral activation, myocardial stretch and injury, and remodelling of the extracellular matrix.^3^, ^4^ These changes can lead to electrophysiological, mechanical, and structural abnormalities.

The use of a combination of different risk markers can provide important, incremental pieces of information for prognostication and potentially can better guide treatment strategies in HF patients. These markers include traditional echocardiographic parameters such as left ventricular (LV) ejection fraction (LVEF) and global longitudinal strain (GLS),^5^ different blood biomarkers,^6^ pro‐inflammatory indices,^7^ prognostic nutritional index (PNI),^8^ frailty status,^9^ and various electrocardiographic (ECG) indices.^10^ While ECG variables reflecting ventricular function are relatively well‐established in terms of their ability to provide prognostic information, those reflecting atrial function are also important predictors of disease outcomes. The latter include ECG P‐wave indices such as P‐wave duration (PWD) and P‐wave dispersion.^11^ Recently, investigators have investigated atrial strain imaging by speckle‐tracking echocardiography (STE) and used these for risk stratification.^12^ In this study, we tested the hypotheses that a combination of atrial echocardiographic strain imaging, P‐wave indices, and inflammatory and nutritional indices can be used for risk stratification in HF and that the predictive power can be further improved by machine learning approaches.

## Methods

### Study population, baseline characteristics, and laboratory information

This study received ethical approval from The Joint Chinese University of Hong Kong—New Territories East Cluster Clinical Research Ethics Committee (G. T. as the principal investigator). It is based on the raw datasets that have already made available by our group in an online repository.^13^, ^14^ Inclusion criteria were adult patients (≥18 years of age) with LVEF ≤ 45% determined by echocardiography between 1 January 2010 and 31 December 2016. The details of patients are linked to a territory‐wide Clinical Data Analysis and Reporting System, with each patient identifiable with a unique reference identifier, enabling access to comprehensive medical records. This has previously been used by local teams for conducting research studies.^15^, ^16^, ^17^

The following clinical details were obtained: age, gender, smoking status, diabetes mellitus, hypercholesterolaemia, coronary artery disease, hypertension, baseline atrial fibrillation (AF), and anti‐hypertensive and anti‐coagulation medications. Complete blood count was performed using automated haematology analysers. Biochemical data such as sodium, potassium, urea, creatinine, and albumin levels were also determined. Neutrophil‐to‐lymphocyte ratio (NLR) was given by the ratio of peripheral neutrophil count/mm^3^ to peripheral lymphocyte count/mm^3^. The PNI was calculated by 10 × serum albumin value (g/dL) + 0.005 × peripheral lymphocyte count/mm^3^.

### Electrocardiographic measurements

P‐wave variables were measured manually from patients who were in sinus rhythm at baseline. Two investigators measured the following parameters from the ECGs: (i) mean PWD was calculated from values obtained from leads V1, II, III, and aVF; (ii) amplitude of the P‐wave was measured from lead V1; (iii) partial or advanced inter‐atrial block (IAB), defined as PWD ≥ 120 ms in the absence or presence of biphasic P‐waves in the inferior leads, respectively; (iv) P‐wave dispersion, defined as the maximum difference in PWD between the leads V1, II, III, and aVF; and (v) P‐wave terminal force in V1 (PTFV1), defined as the area subtended by the terminal negative component of a biphasic P‐wave in lead V1, with the area calculated by multiplication of the duration and depth of the waveform.^18^

### Echocardiography

Comprehensive transthoracic echocardiography was performed using a cardiac ultrasound system with digital storage capacity (E9, General Electric, Milwaukee, WI, USA). Left atrial (LA) diameters, LV diastolic diameters, the thickness of the interventricular septum, and thickness of the LV posterior wall were measured according to the American Society of Echocardiography guidelines. Conventional parasternal short‐axis views at the basal, middle, and apical levels and apical four‐chamber, two‐chamber, and three‐chamber views were obtained. Three consecutive cardiac cycles in sinus rhythm were digitally stored for subsequent analyses. All the images were obtained at a frame rate of 60–90 frames per second.

LA area was measured with planimetry for four‐chamber and two‐chamber views by tracing the endocardial border, excluding the confluence of the pulmonary veins and the LA appendage. LA volume was measured at end‐systole, before P‐wave, and end‐diastole. STE enables quantification of LA function with relatively high fidelity and accuracy.^19^ Importantly, STE strain imaging allows the detection of early LA functional impairment in the absence of LA enlargement. In this study, we evaluated the prevalence of LA enlargement and functional impairment as assessed by the volumetric measurement and deformation indices and investigated the association of LA functional impairment with LA enlargement and with ECG P‐wave indices. Similarly, LV area was measured with planimetry for four‐chamber, three‐chamber and two‐chamber views by tracing the LV endocardial border. GLS of wathe

### Outcomes, logistic regression, and multi‐task learning model

Outcome measures include incident AF, transient ischaemic attack (TIA)/stroke, and all‐cause mortality. Descriptive statistics were presented as number (percentage) or median [interquartile range] as appropriate. Kruskal–Wallis test was applied for comparisons of differences between groups. The relationship between clinical and laboratory parameters and study outcomes was explored using univariate logistic regression. Variables with *P* < 0.10 were further included in a multivariate model. Crude and adjusted odds ratios were reported in 95% confidence intervals. To develop a scoring system, the significant variables in the final multivariate logistic regression model were included. Briefly, a point assigned to a variable would be equivalent to the halved value of adjusted odds ratio, rounded up to the nearest integer. The diagnostic performance of risk variables was evaluated by Harrel's *C*‐statistic and receiver operating characteristic curve using cut‐off values determined by Youden's *J* statistic. The multi‐task learning model consists of common knowledge sharing layers and task‐specific knowledge sharing layers.

### Statistical analysis

Statistical analysis was performed using Stata (Version 13.0). Comparisons between groups were made using Fisher's exact test or Kruskal–Wallis ANOVA for categorical and continuous variables, respectively. A two‐sided *P*‐value < 0.05 was considered statistically significant.

## Results

In our HF cohort (*n* = 312), the median age was 64 [55–73] years, and 235 (75%) were male. The baseline characteristics, laboratory information, and ECG and echocardiographic measurements are shown in *Table 1*. Sodium and potassium ion concentrations took median values of 139.2 [136.0–141.3] and 4.0 [3.7–4.4] mM, respectively. Urea and creatinine were 0.49 [0.36–0.64] and 109 [88–159] mM. Cell counts for neutrophils and lymphocytes were 5.5 [4.3–7.3] and 1.2 [0.9–1.8] per mm^3^, yielding a neutrophil‐to‐lymphocyte ratio (NLR) of 4.41 [2.78–7.82]. Albumin took a median level of 35.1 [30.2–39.0], yielding a PNI [given by 10 × serum albumin value (g/dL) + 0.005 × peripheral lymphocyte count (per mm^3^)] of 35.0 [30.2–39.0]. A total of 22 and 54 patients received implantable cardioverter‐defibrillators.

**Table 1:** Baseline characteristics of heart failure patients included in this study (*n* = 312).

Variables	Value
Male gender	186 (60)
Age	64 (55–73)
Symptoms	
Dyspnoea	237 (76)
Paroxysmal nocturnal dyspnoea	66 (21)
Orthopnoea	130 (42)
Peripheral oedema	160 (51)
Crepitations/wheeze	156 (50)
Smoking	138 (44)
Hypertension	149 (48)
Hypercholesterolaemia	105 (34)
Ischaemic heart disease	159 (51)
Type 2 diabetes mellitus	109 (35)
MS	
None/trace	310 (99)
Mild	0 (0)
Moderate	2 (1)
Severe	0 (0)
MR	
None/trace	40 (13)
Mild	162 (52)
Moderate	83 (27)
Severe	27 (9)
AS	
None/trace	304 (97)
Mild	2 (0.6)
Moderate	5 (2)
Severe	1 (0.3)
AR	
None/trace	196 (63)
Mild	104 (33)
Moderate	11 (4)
Severe	1 (0.3)
TS	
None/trace	0 (0)
Mild	0 (0)
Moderate	0 (0)
Severe	0 (0)
TR	
None/trace	68 (22)
Mild	190 (61)
Moderate	39 (13)
Severe	15 (5)
PS	
None/trace	0 (0)
Mild	0 (0)
Moderate	0 (0)
Severe	0 (0)
PR	
None/trace	205 (66)
Mild	105 (34)
Moderate	1 (0.3)
Severe	1 (0.3)
Left atrial diameter (cm)	4.3 (3.8–4.8)
Left ventricular ejection fraction (%)	30 (22–35)
P‐wave duration (ms)	125 (114–136)
P‐wave dispersion (ms)	31 (21–41)
Abnormal P‐wave terminal force in V1	61 (33–93)
Inter‐atrial block	217 (80%)^a^
Inter‐atrial block type	
None	56 (18)
Partial inter‐atrial block	139 (45)
Advanced inter‐atrial block	45 (14)
Atrial fibrillation	72 (23)
Serum Na^+^	139.2 (136.0–141.3)
Serum K^+^	4.0 (3.7–4.4)
Serum urea	
Serum creatinine	109 (88–159)
Serum albumin	35.1 (30.2–39.0)
MDRD	47.4 (24.9–63.0)
NLR	4.41 (2.78–7.82)
PNI	35.0 (30.2–39.0)
NLR/PNI * 100	13.5 (7.7–23.4)
N2AC score	2 (1–3)
GLS	−10.0% (−12.0 to −8.1)
GLS > −9.25%	119 (41)

Data are presented as median (interquartile range) or number (percentage) as appropriate.

AR, aortic regurgitation; AS, aortic stenosis; GLS, global longitudinal strain; MDRD, Modification of Diet in Renal Disease; MR, mitral regurgitation; MS, mitral stenosis; NLR, neutrophil‐to‐lymphocyte ratio; PNI, prognostic nutritional index; PR, pulmonary regurgitation; PS, pulmonary stenosis; TR, tricuspid regurgitation; TS, tricuspid stenosis.

^a^ Excluding patients in atrial fibrillation.

### Electrocardiographic and echocardiographic measurements

On electrocardiogram findings, 39 patients had AF on admission to our hospital without prior ECGs in sinus rhythm available for analysis. The remaining 273 patients were in sinus rhythm and were analysed further for their P‐wave variables. Maximum PWD, P‐wave dispersion, and PTFV1 took median values of 125 [114–136] ms, 31 [21–41] ms, and 61 [33–93] mm·mV, respectively.

**Figure 1:**
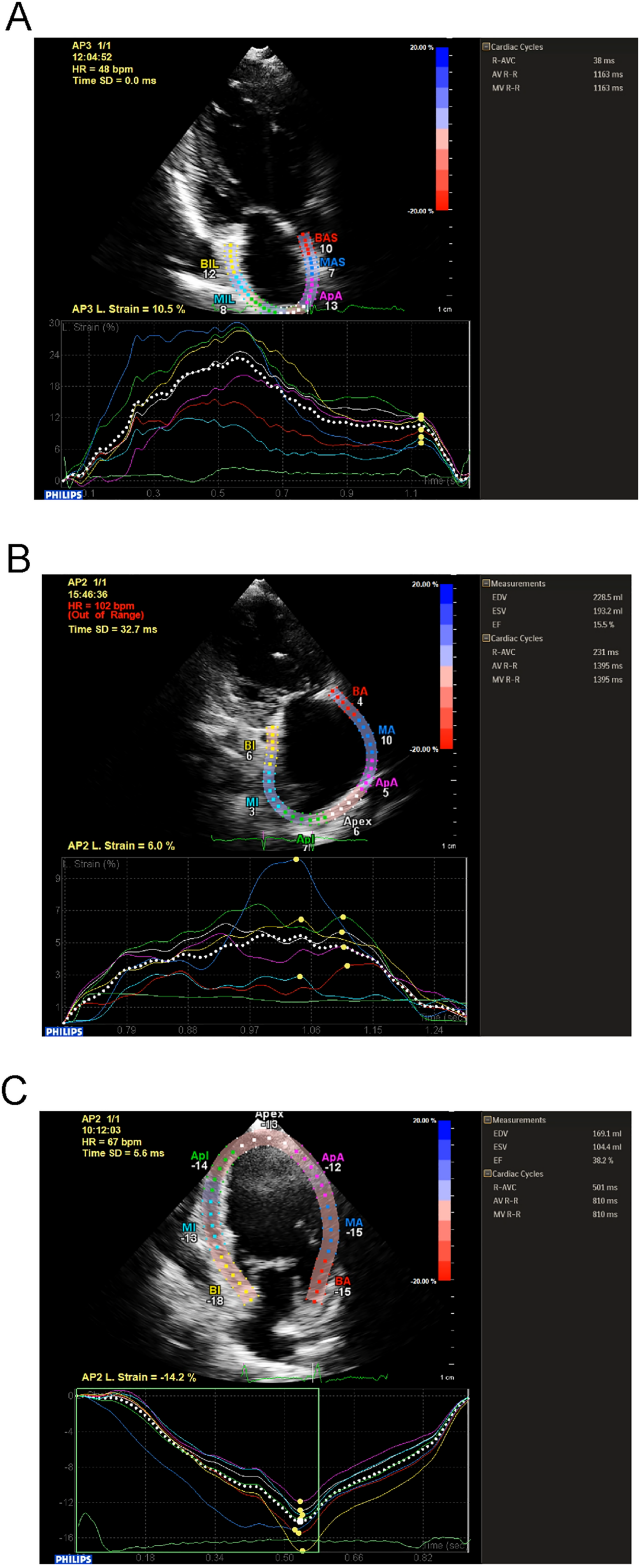
Delineation of the left atrial border for a patient in sinus rhythm and the resulting strain curves showing characteristic three phases two peaks, which reflect peak atrial longitudinal strain (PALS) and peak atrial contraction strain (PACS) (A). Delineation of the left atrial border for a typical patient in atrial fibrillation and the resulting strain curves showing a decrease in PALS and disappearance of the second peak due to a lack of PACS (B). Delineation of the left ventricular border and the resulting strain curves (C).

Echocardiography was used to assess atrial and ventricular function. Tracing of the atrial border was performed manually to determine the region of interest (*Figure 1A*). A typical example of atrial strain curves for a patient in sinus rhythm is shown in *Figure 1B*, with the characteristic three phases with two peaks, reflecting peak atrial longitudinal strain (PALS) and peak atrial contraction strain (PACS), respectively. By contrast, for patients in AF, PALS is reduced, and the second peak disappears because of a lack of PACS (*Figure 1C*). Furthermore, reservoir, conduit, and contractile strain took values of 12.7% [7.8–19.6], 8.0% [5.1–10.9], and 6.2% [2.8–11.2], respectively. LVEF took a median value of 30% [22–35]. Moreover, LV GLS was −10.1% [−12.0 to −8.1] for the overall cohort.

### Predictors of adverse outcomes

The findings of univariate logistic regression of laboratory, ECG, and ventricular echocardiographic variables for new‐onset AF, TIA/stroke, and all‐cause mortality are reported in *Tables S1*–S3, respectively. Those of atrial strain variables are shown in *Tables S4–S6* and of ventricular strain variables are shown in *Tables S7–S9*.

**Figure 2:**
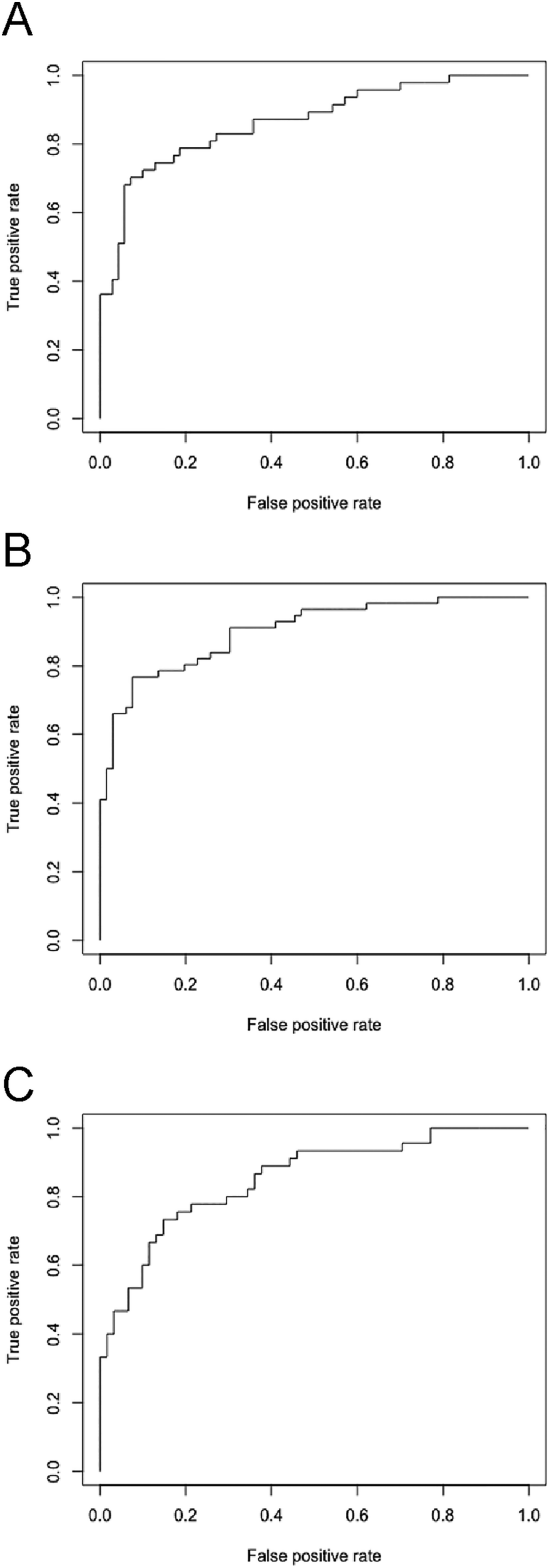
Receiver operating characteristic (ROC) analysis for incident AF (A), TIA/stroke (B) and all‐cause mortality (C). AF, atrial fibrillation; TIA, transient ischaemic attack.

New‐onset AF was associated with age and median LA reservoir and contractile strain (*P* < 0.05). Increased risk of TIA/stroke was associated with age, smoking, type 2 diabetes mellitus, and high N2AC score (*P* < 0.05). Moreover, all‐cause mortality correlated with age, hypertension, type 2 diabetes mellitus, mitral or aortic regurgitation, LA diameter, LVEF, abnormal P‐wave terminal force in V1, partial IAB, chronic kidney disease, urea, creatinine, albumin, high NLR in Quartile 4, PNI, average ventricular GLS, LA reservoir, conduit, and contractile strain (*P* < 0.05). The area under the curve values from receiver operating characteristic analysis for incident AF, stroke, and all‐cause mortality are shown in *Figure 2A–C*.

### Comparative performance for diagnostic ability between logistic regression and machine learning approaches

Variables that achieved *P* < 0.10 in univariate analysis were entered to a multivariate model. A four‐point score (N2AC score) was created, combining NLR at Quartile 4, PNI at Quartiles 1 and 2, age > 62.5 years, and creatinine > 119.5. N2AC scores of 2, 3, and 4 were associated with 3.28 [1.25–8.61], 5.94 [2.20–16.05], and 25.00 [5.48–114.10], respectively, higher odds for all‐cause mortality.

**Figure 3:**
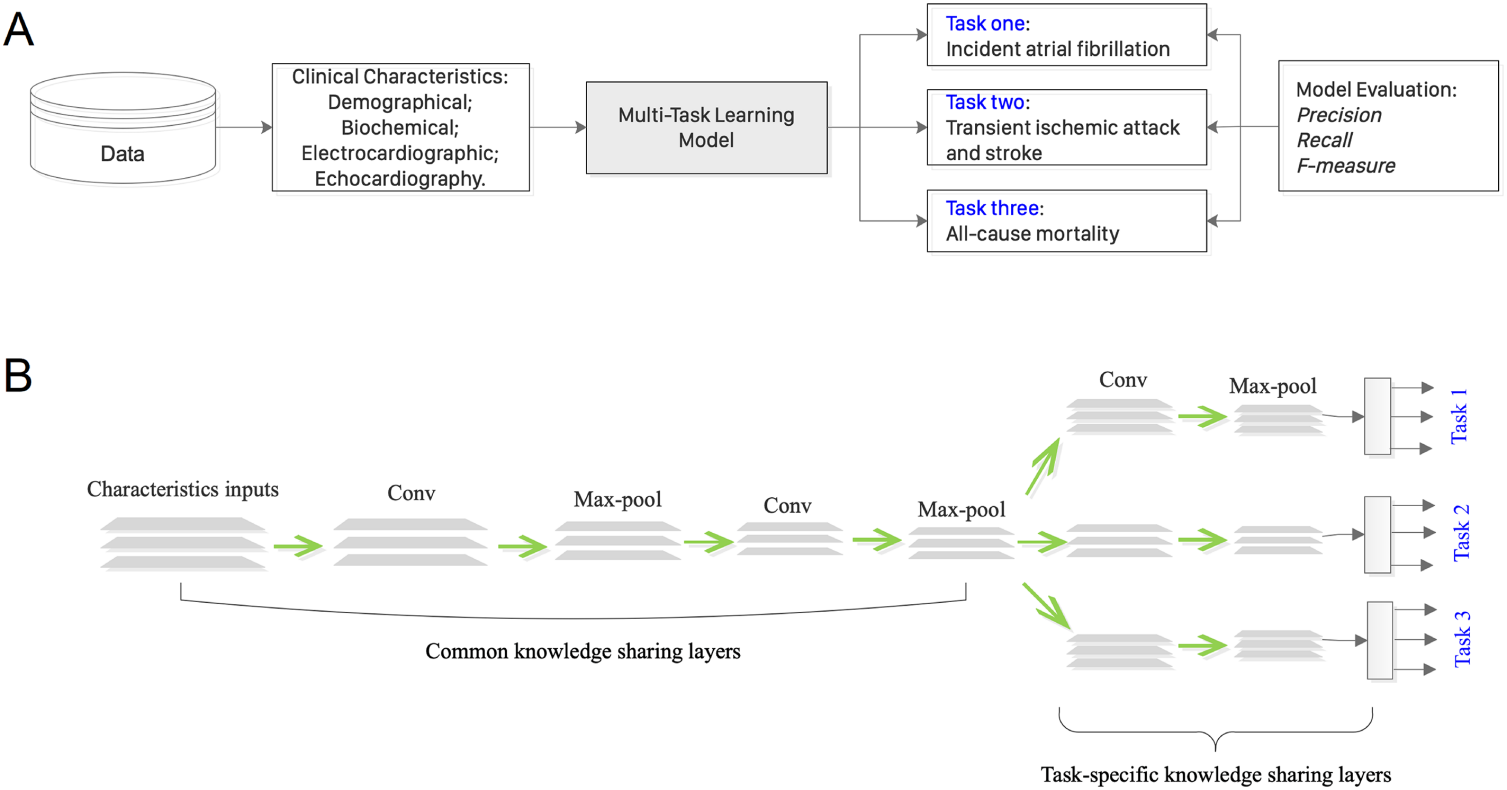
Outcome prediction using a multi‐task learning model. Summary of the analysis undertaken in this study (A). The model consists of common knowledge sharing layers and task‐specific knowledge sharing layers (B).

A convolutional network‐based, multi‐task learning method was then used to predict outcomes of incident AF (Task 1), TIA/stroke (Task 2), and all‐cause mortality (Task 3) (*Figure 3A*). The principles are shown in *Figure 3B* with the model consisting of common knowledge sharing layers and task‐specific knowledge sharing layers. The model was trained with 80% of samples (*n* = 250) while using the remaining 20% (*n* = 62) samples for testing. To better test the model's out‐of‐sample prediction ability, five‐fold cross‐validation was adopted. The computation results are evaluated with several widely used metrics (including recall, precision, and F1 score), as shown in *Table S10*, in which the resulting prediction performance of multi‐task learning was compared with that of multilayer perceptron and multivariable logistic regression model for each individual task prediction. Multi‐task learning outperformed both multilayer perceptron and logistic regression on evaluation metrics.

## Discussion

HF is characterized by a combination of electrical, mechanical, and structural abnormalities. In this study, we tested the hypothesis that machine learning approaches incorporating multi‐modality data can improve risk prediction and stratification as compared with traditional logistic regression analysis. The different data types were cardiac‐specific variables from ECG, traditional and strain echocardiographic imaging, and systemic laboratory variables reflecting renal function, inflammation, and nutritional status.

### Atrial and ventricular electrophysiological, mechanical, and structural abnormalities in heart failure

Conduction delay along the Bachmann bundle between left and right atria leads to IAB.^20^ The association between IAB and supraventricular tachyarrhythmias, such as atrial flutter and AF, is known as Bayés syndrome. HF and AF share common risk factors and have a bidirectional relationship; each predisposes to the other condition.^21^ HF is a major risk factor for AF through atrial dilatation with progressive structural remodelling and interstitial fibrosis, predisposing to re‐entrant activity within the atrium.^22^ Conversely, AF leads to a loss of reservoir, conduit, and booster functions, decreasing cardiac output by up to a quarter and can cause a tachycardia‐induced cardiomyopathy.^23^ These abnormalities can be quantified by ECG and STE. In our cohort, we found a range of atrial electrophysiological abnormalities, from partial to advanced IAB and AF. This may reflect a spectrum of disease severity. We also found increased LA diameter and decreased atrial reservoir, conduit, and contractile strain. The inter‐relationship between atrial electrical and mechanical remodelling, development of IAB, stroke, and mortality events remains less explored. In our study, PWD, partial IAB, or advanced IAB did not predict new‐onset AF or stroke. By contrast, other groups have found advanced IAB to be a significant predictor of incident AF and stroke in a general HF cohort.^24^ Furthermore, amplified PWDs were shown to predict incident AF in patients with HF with preserved ejection fraction.^25^ In terms of echocardiography, abnormal LA strain has demonstrated prognostic value in both HF with preserved ejection fraction^26^ as well as reduced ejection fraction.^27^ Moreover, chronic HF patients with reduced PALS showed a higher frequency of AF development, suggesting a close relationship between mechanical and electrical dysfunction.^28^

Moreover, LV function is a classical determinant of adverse outcomes and can be examined using echocardiography. The primary measure is the LVEF, the fraction of ventricular volume ejected per beat. A seminal trial of 7599 patients found that from LVEF of 45%, a 10% decrease led to a 39% increase in all‐cause mortality in HF.^29^ LVEF is also a significant predictor of sudden cardiac death.^30^ Furthermore, LV strain from myocardial deformation imaging has offered additional value for risk stratification.^31^ For example, a multi‐centre study found that GLS had the highest prognostic value and the highest combination of sensitivity and specificity for prediction of adverse events, and outperformed LVEF alone.^32^

### Inflammatory and nutrition indices

Different laboratory indices quantifying the level of inflammation have been investigated for their ability to predict adverse outcomes. A popular index is the NLR, which reflects the balance between pro‐inflammatory and anti‐inflammatory patterns, given by increased neutrophils and reduced lymphocytes, respectively.^33^ NLR has previously been identified as a significant predictor of long‐term outcomes in acute decompensated HF.^34^ Indeed, a recent meta‐analysis found that raised NLR was significantly associated with a higher likelihood of all‐cause mortality.^35^ In this study, NLR was found to predict all‐cause mortality in our cohort of HF patients with LVEF ≤ 45%. NLR can also predict adverse outcomes in other cardiac diseases such as mitral regurgitation^36^ and aortic stenosis.^37^

Moreover, poor nutrition leads to impaired immune responses and leads to adverse outcomes in HF.^38^ A comprehensive dietary and nutritional assessment enable a personalized approach to optimize health in cardiac patients. However, their routine performance is limited by the lack of time in daily clinical practice. Thus, laboratory‐based indices for nutrition have been proposed to aid rapid assessment and risk stratification. An example is the PNI, which is given by [(10 × serum albumin (g/dL)) + (0.005 × total lymphocyte count)].^39^ It is convenient to calculate, as it does not require manual evaluation of body mass or height. In our study, we found that low albumin and N2AC score were associated with a higher incidence of stroke. Although PNI was not a significant predictor of TIA/stroke, PNI at Quartiles 3 and 4 was nevertheless associated with lower mortality compared to Quartile 1. These findings indicate that PNI can be used as a surrogate marker for nutritional status and can be used to identify HF patients with high mortality.

### Machine learning approaches

Machine learning techniques have been successfully used to investigate cardiovascular diseases by exploiting complex and informative interactions among dependent variables from clinical and behavioural data. Machine learning‐based methods achieved high accurate results on the basis of large sample sizes of training datasets. For instance, deep learning models for HF prediction that are constructed on neural networks usually require large‐scale labelled samples to train neural networks with a huge number of model parameters. However, in some real‐life applications, this requirement cannot be fulfilled because large‐scale (labelled) samples are difficult to obtain. For instance, we only have 312 patients in our HF dataset. In this case, shallow learning models, let alone deep models, are constrained by limited training samples. To overcome this data insufficiency problem, multi‐task learning^40^ has been recognized as a good solution to gain overall prediction performance improvement when multiple related tasks are jointly learned, although each task has limited training samples. Multi‐task learning is inspired by the fact that human apply knowledge gained from previous tasks to help us learn a new task. There is no distinction among multiple tasks, and the ultimate objective is to improve the learning performance of all tasks. Multi‐task learning has been successfully used across many applications, such as cardiac image analysis^41^.

In this study, we had three prediction tasks: incident AF (Task 1), TIA/stroke (Task 2), and all‐cause mortality (Task 3), and the three tasks are related to each other. We used a convolutional network‐based multi‐task learning method, in which we design a common knowledge sharing layer and a task‐specific knowledge sharing layer to jointly capture the important relevant information among the three prediction tasks. The deep multi‐task learning method is not sensitive to data insufficiency and can improve the overall performance of all three prediction tasks. The experimental results of our study demonstrate that the deep multi‐task learning model is an efficient approach to jointly achieve overall performance improvement for the prediction of multiple tasks/outcomes by exploiting complex and informative interactions among dependent variables in HF.

### Strengths and limitations

The main strength of our study is the relatively large sample size involving >300 patients with comprehensive ECG and echocardiographic measurements supplemented by laboratory data. However, it is limited by the single ethnicity of the study subjects. Future studies are needed to validate our findings and models in external cohorts involving other ethnicities. Moreover, it is well‐recognized that HF with mid‐range EF (HFmEF) may have a different disease course compared to HF with reduced EF patients, and our sample size for the HFmEF group was small. The latter group will need to be examined separately in the future. Finally, the convolutional network‐based multi‐task learning method we use provides highly accurate risk predictions, but it fails to generate the importance ranking of specific parameters among clinical, demographic, ECGs, and so forth for the predictions. Therefore, it is of great interest to develop an interpretable deep multi‐task learning model for risk stratification in HF.

## Conclusions

Multi‐modality assessment using baseline variables, laboratory, ECG, and echocardiographic indices is important for risk stratification in HF. A machine learning approach provides additional value for improving outcome prediction.

## Conflict of interest

None declared.

## Funding

This study was supported by Theme‐based Research Scheme project of the Research Grants Council of Hong Kong to QZ (T32‐102/14‐N).

## Supporting information


**Table S1.** Univariate analysis for new onset atrial fibrillation.
**Table S2.** Univariate analysis for transient ischemic attack (TIA)/stroke.
**Table S3.** Univariate analysis for all‐cause mortality.
**Table S4.** Univariate analysis of atrial strain variables for new onset atrial fibrillation.
**Table S5.** Univariate analysis of atrial strain variables for transient ischemic attack (TIA)/stroke.
**Table S6.** Univariate analysis of atrial strain variables for mortality.
**Table S7.** Univariate analysis of ventricular strain variables for new onset atrial fibrillation.
**Table S8.** Univariate analysis of ventricular strain variables for TIA/stroke.
**Table S9.** Univariate analysis of ventricular strain variables for all‐cause mortality.
**Table S10.** Comparative of diagnostic performance between logistic regression, multilayer perceptron and multi‐task learning
