# Supplementary material for: Multi‐modality machine learning approach for risk stratification in heart failure with left ventricular ejection fraction ≤ 45%
Source: ESC Heart Fail. 2020 Oct 23;7(6):3716–25. doi: 10.1002/ehf2.12929 (PMC7754744; doi:10.1002/ehf2.12929)
Supplement: Supplementary file 1 — Table S1. Univariate analysis for new onset atrial fibrillation. Table S2. Univariate analysis for transient ischemic attack (TIA)/stroke. Table S3. Univariate analysis for all‐cause mortality. Table S4. Univariate analysis of atrial strain variables for new onset atrial fibrillation. Table S5. Univariate analysis of atrial strain variables for transient ischemic attack (TIA)/stroke. Table S6. Univariate analysis of atrial strain variables for mortality. Table S7. Univariate analysis of ventricular strain variables for new onset atrial fibrillation. Table S8. Univariate analysis of ventricular strain variables for TIA/stroke. Table S9. Univariate analysis of ventricular strain variables for all‐cause mortality. Table S10. Comparative of diagnostic performance between logistic regression, multilayer perceptron and multi‐task learning [file EHF2-7-3716-s001.docx]

**Supplementary Appendix**

*Subgroup analysis based on atrial conduction status*

The maximum PWD for the normal inter-atrial conduction, partial IAB and advanced IAB were 114 [105-117], 128 [117-137], 135 [130-142] ms, with significant differences between groups (P=0.0001). For P-wave dispersion, the values were 27 [18-36], 31 [21-41], 36 [27-44], respectively (P=0.008). For PTFV1, these were 47 [30-87], 61 [33-93] and 74 [38-102] ms.mV, respectively (P>0.05).

On atrial strain parameters, reservoir strain took a value of 19.6 [10.6-28.1], 13.9 [9.9-19.3], 12.0 [8.5-16.6] and 10.7 [5.6-15.1] for the normal inter-atrial conduction, partial IAB, advanced IAB and AF groups, respectively (Kruskal-Wallis test, P=0.0003). For conduit strain, the values were 8.8 [5.5-14.0], 8.2 [4.9-10.9], 6.0 [4.2-8.6] and 8.6 [6.1-11.0] (P=0.01). For contractile strain, they were 10.2 [4.0-15.8], 6.1 [2.6-11.3], 5.8 [2.1-8.8] and 5.0 [2.6-8.8], respectively (P=0.04).

Regarding left ventricular ejection fraction, median values of 30 [22-36], 28 [21-35], 30 [23-35] and 30 [24-35] were observed for normal inter-atrial conduction, partial IAB, advanced IAB and AF groups, respectively, with no significant difference between these groups (P=0.36).

For left ventricular global longitudinal strain, median values of -11.1 [-13.8 to -7.6] for the normal inter-atrial conduction group, -9.8 [-11.7 to -7.8] for the partial IAB group, -10.0 [-12.1 to -8.2] for the advanced IAB group and -10.0 [-12.0 to -8.3] for the AF group were observed. No significant between the groups was observed (P > 0.05).

**Supplementary Table 1. Univariate analysis for new onset atrial fibrillation.**

|  | | | | New onset atrial fibrillation | | | | | | |  |
| --- | --- | --- | --- | --- | --- | --- | --- | --- | --- | --- | --- |
| Variables | | |  | | | Crude Odds Ratio (95% CI) | | | P-value |  |  |
| Male gender |  | 0.99 (0.53-1.85) | | | | | | 0.979 | |  |  |
| Age |  | 1.03 (1.01-1.06) | | | | | | **0.003** | |  |  |
| *Symptoms* |  |  | | | | | |  | |  |  |
| Dyspnoea |  | 1.15 (0.59-2.25) | | | | | | 0.688 | |  |  |
| Paroxysmal nocturnal dyspnoea |  | 0.92 (0.46-1.84) | | | | | | 0.807 | |  |  |
| Orthopnoea |  | 1.07 (0.61-1.88) | | | | | | 0.807 | |  |  |
| Peripheral oedema |  | 1.28 (0.74-2.24) | | | | | | 0.379 | |  |  |
| Crepitations/wheeze |  | 1.34 (0.77-2.33) | | | | | | 0.307 | |  |  |
| Smoking |  | 1.42 (0.80-2.51) | | | | | | 0.229 | |  |  |
| Hypertension |  | 1.74 (0.97-3.11) | | | | | | 0.062 | |  |  |
| Hypercholesterolaemia | | |  | | 1.54 (0.87-2.71) | | 0.137 | | | | |
| Ischaemic heart disease | | |  | | 1.24 (0.69-2.23) | | 0.470 | | | | |
| Type 2 diabetes mellitus | | |  | | 1.11 (0.63-1.94) | | 0.720 | | | | |
| *MR* | | |  | |  | |  | | | | |
| None/trace | | |  | | 1 (reference) | | - | | | | |
| Mild | | |  | | 2.75 (0.79-9.62) | | 0.113 | | | | |
| Moderate | | |  | | 3.86 (1.05-14.22) | | **0.043** | | | | |
| Severe | | |  | | 6.84 (1.65-28.38) | | **0.008** | | | | |
| *AS* | | |  | |  | |  | | | | |
| None/trace | | |  | | 1 (reference) | | - | | | | |
| Mild | | |  | | 2.98 (0.18-48.40) | | 0.442 | | | | |
| Moderate | | |  | | - (empty) | | - | | | | |
| Severe | | |  | | - (empty) | | - | | | | |
| *AR* | | |  | |  | |  | | | | |
| None/trace | | |  | | 1 (reference) | | - | | | | |
| Mild | | |  | | 0.86 (0.47-1.58) | | 0.624 | | | | |
| Moderate | | |  | | 2.44 (0.63-9.52) | | 0.199 | | | | |
| Severe | | |  | | - (empty) | | - | | | | |
| *TR* | | |  | |  | |  | | | | |
| None/trace | | |  | | 1 (reference) | | - | | | | |
| Mild | | |  | | 1.85 (0.83-4.09) | | 0.130 | | | | |
| Moderate | | |  | | 2.25 (0.82-6.15) | | 0.115 | | | | |
| Severe | | |  | | 1.39 (0.32-6.02) | | 0.656 | | | | |
| *PR* | | |  | |  | |  | | | | |
| None/trace | | |  | | 1 (reference) | | - | | | | |
| Mild | | |  | | 1.37 (0.77-2.44) | | 0.283 | | | | |
| Moderate | | |  | | - (empty) | | - | | | | |
| Severe | | |  | | - (empty) | | - | | | | |
| Left atrial diameter (cm) | | |  | | 1.49 (0.97-2.27) | | 0.067 | | | | |
| Ejection fraction (%) | | |  | | 1.02 (0.98-1.05) | | 0.370 | | | | |
| P-wave duration (ms) | | |  | | 0.99 (0.97-1.01) | | 0.189 | | | | |
| P-wave dispersion (ms) | | |  | | 0.99 (0.97-1.01) | | 0.279 | | | | |
| P-wave terminal force in V1 | | |  | | 0.65 (0.37-1.16) | | 0.142 | | | | |
| Interatrial block | | |  | | 1.42 (0.69-2.94) | | 0.341 | | | | |
| *Interatrial block type* | | |  | |  | |  | | | | |
| None | | |  | | 1 (reference) | | N/A | | | | |
| Partial interatrial block | | |  | | 1.45 (0.69-3.04) | | 0.327 | | | | |
| Advanced interatrial block | | |  | | 1.32 (0.51-3.41) | | 0.562 | | | | |
| Serum Na^+^ | | |  | | 0.99 (0.93-1.05) | | 0.724 | | | | |
| Serum K^+^ | | |  | | 0.94 (0.61-1.46) | | 0.789 | | | | |
| Serum urea | | |  | | 1.02 (0.98-1.05) | | 0.423 | | | | |
| Serum creatinine | | |  | | 1.00 (1.00-1.00) | | 0.644 | | | | |
| Serum albumin | | |  | | 0.99 (0.94-1.03) | | 0.578 | | | | |
| MDRD | | |  | | 0.96 (0.92-1.00) | | **0.043** | | | | |
| *NLR Quartiles* | | |  | |  | |  | | | | |
| Quartile 1 | | |  | | 1 (reference) | | N/A | | | | |
| Quartile 2 | | |  | | 1.38 (0.61-3.13) | | 0.443 | | | | |
| Quartile 3 | | |  | | 0.91 (0.39-2.13) | | 0.822 | | | | |
| Quartile 4 | | |  | | 1.07 (0.47-2.46) | | 0.871 | | | | |
| *PNI Quartiles* | | |  | |  | |  | | | | |
| Quartile 1 | | |  | | 1 (reference) | | N/A | | | | |
| Quartile 2 | | |  | | 1.18 (0.53-2.63) | | 0.678 | | | | |
| Quartile 3 | | |  | | 0.90 (0.40-2.03) | | 0.804 | | | | |
| Quartile 4 | | |  | | 0.73 (0.31-1.74) | | 0.474 | | | | |
| NLR/PNI | | |  | | 0.998 (0.99-1.01) | | 0.590 | | | | |
| *N2AC score* | | |  | |  | |  | | | | |
| 0 | | |  | | 1 (reference) | | N/A | | | | |
| 1 | | |  | | 0.92 (0.32-2.65) | | 0.882 | | | | |
| 2 | | |  | | 1.42 (0.51-3.96) | | 0.499 | | | | |
| 3 | | |  | | 1.47 (0.52-4.18) | | 0.470 | | | | |
| 4 | | |  | | 3.56 (0.96-13.13) | | 0.057 | | | | |
| GLS | | |  | | 1.03 (0.92-1.14) | | 0.655 | | | | |
| GLS > -9.25% | | |  | | 1.09 (0.61-1.97) | | 0.762 | | | | |

**Supplementary Table 2. Univariate analysis for transient ischemic attack (TIA)/stroke.**

|  | | | TIA/Stroke | | | | | |  |
| --- | --- | --- | --- | --- | --- | --- | --- | --- | --- |
| Variables | | |  | | Crude Odds Ratio (95% CI) | | | P-value |  |
| Male gender |  | 1.58 (0.78-3.22) | | | | | 0.207 | |  |
| Age |  | 1.04 (1.01-1.06) | | | | | **0.002** | |  |
| *Symptoms* |  |  | | | | |  | |  |
| Dyspnoea |  | 1.22 (0.61-2.44) | | | | | 0.583 | |  |
| Paroxysmal nocturnal dyspnoea |  | 1.18 (0.60-2.31) | | | | | 0.632 | |  |
| Orthopnoea |  | 1.10 (0.62-1.95) | | | | | 0.748 | |  |
| Peripheral oedema |  | 1.22 (0.69-2.16) | | | | | 0.496 | |  |
| Crepitations/wheeze |  | 1.68 (0.94-3.00) | | | | | 0.078 | |  |
| Smoking |  |  | | | | |  | |  |
| Hypertension |  |  | | | | |  | |  |
| Hypercholesterolaemia | | |  |  | |  | | | |
| Ischaemic heart disease | | |  |  | |  | | | |
| Type 2 diabetes mellitus | | |  |  | |  | | | |
| *MS* | | |  |  | |  | | | |
| None/trace | | |  | 1 (reference) | | - | | | |
| Mild | | |  | - (collinearity) | | - | | | |
| Moderate | | |  | - (empty) | | - | | | |
| Severe | | |  | - (empty) | | - | | | |
| *MR* | | |  |  | |  | | | |
| None/trace | | |  | 1 (reference) | | - | | | |
| Mild | | |  |  | |  | | | |
| Moderate | | |  |  | |  | | | |
| Severe | | |  |  | |  | | | |
| *AS* | | |  |  | |  | | | |
| None/trace | | |  | 1 (reference) | | - | | | |
| Mild | | |  | - (collinearity) | | - | | | |
| Moderate | | |  | - (empty) | | - | | | |
| Severe | | |  | - (empty) | | - | | | |
| *AR* | | |  |  | |  | | | |
| None/trace | | |  | 1 (reference) | | - | | | |
| Mild | | |  |  | |  | | | |
| Moderate | | |  |  | |  | | | |
| Severe | | |  | - (empty) | | - | | | |
| *TR* | | |  |  | |  | | | |
| None/trace | | |  | 1 (reference) | | - | | | |
| Mild | | |  |  | |  | | | |
| Moderate | | |  |  | |  | | | |
| Severe | | |  |  | |  | | | |
| *PR* | | |  |  | |  | | | |
| None/trace | | |  | 1 (reference) | | - | | | |
| Mild | | |  |  | |  | | | |
| Moderate | | |  | - (empty) | | - | | | |
| Severe | | |  | - (empty) | | - | | | |
| Left atrial diameter (cm) | | |  |  | |  | | | |
| Ejection fraction (%) | | |  |  | |  | | | |
| P-wave duration (ms) | | |  |  | |  | | | |
| P-wave dispersion (ms) | | |  |  | |  | | | |
| P-wave terminal force in V1 | | |  |  | |  | | | |
| Interatrial block | | |  |  | |  | | | |
| *Interatrial block type* | | |  |  | |  | | | |
| None | | |  | 1.00 (reference) | | N/A | | | |
| Partial interatrial block | | |  |  | |  | | | |
| Advanced interatrial block | | |  |  | |  | | | |
| New onset atrial fibrillation | | |  |  | |  | | | |
| Serum Na^+^ | | |  |  | |  | | | |
| Serum K^+^ | | |  |  | |  | | | |
| Serum urea | | |  |  | |  | | | |
| Serum creatinine | | |  |  | |  | | | |
| Serum albumin | | |  |  | |  | | | |
| MDRD | | |  |  | |  | | | |
| NLR Quartiles | | |  |  | |  | | | |
| Quartile 1 | | |  | 1 (reference) | | N/A | | | |
| Quartile 2 | | |  |  | |  | | | |
| Quartile 3 | | |  |  | |  | | | |
| Quartile 4 | | |  |  | |  | | | |
| PNI Quartiles | | |  |  | |  | | | |
| Quartile 1 | | |  | 1 (reference) | | N/A | | | |
| Quartile 2 | | |  |  | |  | | | |
| Quartile 3 | | |  |  | |  | | | |
| Quartile 4 | | |  |  | |  | | | |
| NLR/PNI | | |  |  | |  | | | |
| N2AC score | | |  |  | |  | | | |
| 0 | | |  | 1 (reference) | | N/A | | | |
| 1 | | |  |  | |  | | | |
| 2 | | |  |  | |  | | | |
| 3 | | |  |  | |  | | | |
| 4 | | |  |  | |  | | | |
| GLS | | |  |  | |  | | | |
| GLS > -9.25% | | |  |  | |  | | | |

**Supplementary Table 3. Univariate analysis for all-cause mortality.**

|  | | | Mortality | | | | | |  |
| --- | --- | --- | --- | --- | --- | --- | --- | --- | --- |
| Variables | | |  | | Crude Odds Ratio (95% CI) | | | P-value |  |
| Male gender |  | 1.17 (0.69-2.01) | | | | | 0.559 | |  |
| Age |  | 1.06 (1.03-1.08) | | | | | **<0.001** | |  |
| *Symptoms* |  |  | | | | |  | |  |
| Dyspnoea |  | 2.15 (1.19-3.89) | | | | | 0.012 | |  |
| Paroxysmal nocturnal dyspnoea |  | 1.58 (0.91-2.74) | | | | | 0.104 | |  |
| Orthopnoea |  | 1.58 (1.00-2.52) | | | | | 0.052 | |  |
| Peripheral oedema |  | 2.03 (1.27-3.25) | | | | | **0.003** | |  |
| Crepitations/wheeze |  | 2.09 (1.31-3.34) | | | | | **0.002** | |  |
| Smoking |  | 0.99 (0.62-1.58) | | | | | 0.962 | |  |
| Hypertension |  | 2.55 (1.55-4.17) | | | | | **<0.001** | |  |
| Hypercholesterolaemia | | |  | 1.07 (0.69-1.73) | | 0.766 | | | |
| Ischaemic heart disease | | |  | 1.55 (0.95-2.54) | | 0.077 | | | |
| Type 2 diabetes mellitus | | |  | 1.80 (1.13-2.88) | | **0.013** | | | |
| *MS* | | |  |  | |  | | | |
| None/trace | | |  | 1 (reference) | | - | | | |
| Mild | | |  | 1.63 (0.10-26.39) | | 0.729 | | | |
| Moderate | | |  | - (empty) | | - | | | |
| Severe | | |  | - (empty) | | - | | | |
| *MR* | | |  |  | |  | | | |
| None/trace | | |  | 1 (reference) | | - | | | |
| Mild | | |  | 1.56 (0.66-3.69) | | 0.309 | | | |
| Moderate | | |  | 2.64 (1.07-6.53) | | **0.036** | | | |
| Severe | | |  | 4.54 (1.50-13.74) | | **0.007** | | | |
| *AS* | | |  |  | |  | | | |
| None/trace | | |  | 1 (reference) | | - | | | |
| Mild | | |  | 1.70 (0.11-27.45) | | 0.709 | | | |
| Moderate | | |  | 6.80 (0.75-61.61) | | 0.088 | | | |
| Severe | | |  | - (empty) | | - | | | |
| *AR* | | |  |  | |  | | | |
| None/trace | | |  | 1 (reference) | | - | | | |
| Mild | | |  | 1.65 (1.01-2.69) | | **0.046** | | | |
| Moderate | | |  | 1.67 (0.49-5.67) | | 0.414 | | | |
| Severe | | |  | - (empty) | | - | | | |
| *TR* | | |  |  | |  | | | |
| None/trace | | |  | 1 (reference) | | - | | | |
| Mild | | |  | 1.54 (0.82-2.91) | | 0.179 | | | |
| Moderate | | |  | 2.29 (0.99-5.28) | | 0.053 | | | |
| Severe | | |  | 1.89 (0.91-9.22) | | 0.073 | | | |
| *PR* | | |  |  | |  | | | |
| None/trace | | |  | 1 (reference) | | - | | | |
| Mild | | |  | 1.32 (0.81-2.14) | | 0.263 | | | |
| Moderate | | |  | - (empty) | | - | | | |
| Severe | | |  | - (empty) | | - | | | |
| Left atrial diameter (cm) | | |  | 1.46 (1.03-2.07) | | **0.032** | | | |
| Ejection fraction (%) | | |  | 0.97 (0.94-1.00) | | **0.046** | | | |
| P-wave duration (ms) | | |  | 0.99 (0.97-1.00) | | 0.202 | | | |
| P-wave dispersion (ms) | | |  | 1.00 (0.98-1.01) | | 0.747 | | | |
| P-wave terminal force in V1 | | |  | 2.48 (1.42-4.33) | | **0.001** | | | |
| *Interatrial block* | | |  | 1.91 (1.00-3.66) | | 0.052 | | | |
| *Interatrial block type* | | |  |  | |  | | | |
| None | | |  | 1 (reference) | | N/A | | | |
| Partial interatrial block | | |  | 2.18 (1.12-4.24) | | **0.022** | | | |
| Advanced interatrial block | | |  | 1.11 (0.46-2.66) | | 0.814 | | | |
| New onset atrial fibrillation | | |  | 1.02 (0.72-1.46) | | 0.900 | | | |
| Serum Na^+^ | | |  | 0.95 (0.91-1.00) | | 0.075 | | | |
| Serum K^+^ | | |  | 0.71 (0.48-1.05) | | 0.088 | | | |
| Serum urea | | |  | 1.13 (1.07-1.19) | | **<0.001** | | | |
| Serum creatinine | | |  | 1.01 (1.00-1.01) | | **<0.001** | | | |
| Serum albumin | | |  | 0.91 (0.88-0.95) | | **<0.001** | | | |
| MDRD | | |  | 0.97 (0.95-1.00) | | 0.084 | | | |
| NLR Quartiles | | |  |  | |  | | | |
| Quartile 1 | | |  | 1 (reference) | | N/A | | | |
| Quartile 2 | | |  | 1.22 (0.60-2.50) | | 0.584 | | | |
| Quartile 3 | | |  | 1.62 (0.80-3.30) | | 0.176 | | | |
| Quartile 4 | | |  | 2.36 (1.16-4.81) | | **0.018** | | | |
| PNI Quartiles | | |  |  | |  | | | |
| Quartile 1 | | |  | 1 (reference) | | N/A | | | |
| Quartile 2 | | |  | 0.64 (0.32-1.28) | | 0.203 | | | |
| Quartile 3 | | |  | 0.33 (0.16-0.67) | | **0.002** | | | |
| Quartile 4 | | |  | 0.26 (0.13-0.55) | | **<0.001** | | | |
| NLR/PNI | | |  | 1.01 (1.00-1.03) | | **0.021** | | | |
| N2AC score | | |  |  | |  | | | |
| 0 | | |  | 1 (reference) | | N/A | | | |
| 1 | | |  | 1.45 (0.53-3.97) | | 0.471 | | | |
| 2 | | |  | 3.28 (1.25-8.62) | | **0.016** | | | |
| 3 | | |  | 6.09 (2.56-16.46) | | **<0.001** | | | |
| 4 | | |  | 25.00 (5.48-114.10) | | **<0.001** | | | |
| GLS | | |  | 1.13 (1.03-1.24) | | **0.007** | | | |
| GLS > -9.25% | | |  | 1.95 (1.20-3.17) | | **0.007** | | | |

**Supplementary Table 4. Univariate analysis of atrial strain variables for new onset atrial fibrillation.**

|  | | | | Atrial Fibrillation | | | | |
| --- | --- | --- | --- | --- | --- | --- | --- | --- |
| Variables | | |  | | Univariate Odds Ratio | | P-value |  |
| **Atrial Strain, AP4** |  |  | | | |  | |  |
| Reservoir Strain |  | 1.00 (0.99-1.01) | | | | 0.421 | |  |
| Contractile Strain |  | 0.95 (0.90-1.00) | | | | **0.032** | |  |
| Conduit Strain |  | 0.98 (0.92-1.04) | | | | 0.442 | |  |
| **Atrial Strain, AP3** |  |  | | | |  | |  |
| Reservoir Strain |  | 0.94 (0.90-0.97) | | | | **0.001** | |  |
| Contractile Strain |  | 0.92 (0.87-0.97) | | | | **0.003** | |  |
| Conduit Strain |  | 1.00 (0.95-1.05) | | | | 0.953 | |  |
| **Atrial Strain, AP2** |  |  | | | |  | |  |
| Reservoir Strain |  | 0.94 (0.91-0.97) | | | | **0.001** | |  |
| Contractile Strain |  | 0.95 (0.91-0.99) | | | | **0.026** | |  |
| Conduit Strain |  | 1.04 (0.98-1.10) | | | | 0.235 | |  |
| **Average Atrial Strain** |  |  | | | |  | |  |
| Reservoir Strain |  | 0.93 (0.90-0.97) | | | | **0.001** | |  |
| Contractile Strain |  | 0.94 (0.89-0.99) | | | | **0.026** | |  |
| Conduit Strain |  | 1.02 (0.95-1.09) | | | | 0.573 | |  |

**Supplementary Table 5. Univariate analysis of atrial strain variables for transient ischemic attack (TIA)/stroke.**

|  | | | | TIA/Stroke | | | | |
| --- | --- | --- | --- | --- | --- | --- | --- | --- |
| Variables | | |  | | Univariate Odds Ratio | | P-value |  |
| **Atrial Strain, AP4** |  |  | | | |  | |  |
| Reservoir Strain |  | 0.99 (0.95-1.02) | | | | 0.379 | |  |
| Contractile Strain |  | 0.99 (0.94-1.04) | | | | 0.804 | |  |
| Conduit Strain |  | 0.97 (0.91-1.03) | | | | 0.338 | |  |
| **Atrial Strain, AP3** |  |  | | | |  | |  |
| Reservoir Strain |  | 0.98 (0.95-1.02) | | | | 0.327 | |  |
| Contractile Strain |  | 0.96 (0.91-1.01) | | | | 0.153 | |  |
| Conduit Strain |  | 0.99 (0.94-1.05) | | | | 0.779 | |  |
| **Atrial Strain, AP2** |  |  | | | |  | |  |
| Reservoir Strain |  | 0.97 (0.94-1.01) | | | | 0.105 | |  |
| Contractile Strain |  | 0.98 (0.94-1.03) | | | | 0.420 | |  |
| Conduit Strain |  | 0.92 (0.86-1.00) | | | | **0.039** | |  |
| **Average Atrial Strain** |  |  | | | |  | |  |
| Reservoir Strain |  | 0.98 (0.94-1.01) | | | | 0.214 | |  |
| Contractile Strain |  | 0.99 (0.94-1.04) | | | | 0.620 | |  |
| Conduit Strain |  | 0.94 (0.87-1.01) | | | | 0.124 | |  |

|  | | | | Mortality | | | | |
| --- | --- | --- | --- | --- | --- | --- | --- | --- |
| Variables | | |  | | Univariate Odds Ratio | | P-value |  |
| **Atrial Strain, AP4** |  |  | | | |  | |  |
| Reservoir Strain |  | 1.00 (0.99-1.01) | | | | 0.430 | |  |
| Contractile Strain |  | 0.95 (0.91-0.98) | | | | **0.014** | |  |
| Conduit Strain |  | 0.95 (0.91-1.00) | | | | 0.070 | |  |
| **Atrial Strain, AP3** |  |  | | | |  | |  |
| Reservoir Strain |  | 0.97 (0.94-1.00) | | | | **0.026** | |  |
| Contractile Strain |  | 0.95 (0.91-0.99) | | | | **0.024** | |  |
| Conduit Strain |  | 0.95 (0.90-1.00) | | | | 0.056 | |  |
| **Atrial Strain, AP2** |  |  | | | |  | |  |
| Reservoir Strain |  | 0.96 (0.93-0.99) | | | | **0.007** | |  |
| Contractile Strain |  | 0.96 (0.92-0.99) | | | | **0.019** | |  |
| Conduit Strain |  | 0.95 (0.89-1.00) | | | | 0.069 | |  |
| **Average Atrial Strain** |  |  | | | |  | |  |
| Reservoir Strain |  | 0.96 (0.93-0.99) | | | | **0.008** | |  |
| Contractile Strain |  | 0.94 (0.90-0.98) | | | | **0.006** | |  |
| Conduit Strain |  | 0.93 (0.88-1.00) | | | | **0.036** | |  |

**Supplementary Table 6. Univariate analysis of atrial strain variables for mortality.**

**Table 7. Univariate analysis of ventricular strain variables for new onset atrial fibrillation.**

|  | | | | Atrial Fibrillation | | | | |
| --- | --- | --- | --- | --- | --- | --- | --- | --- |
| Variables | | |  | | Univariate Odds Ratio | | P-value |  |
| Ventricular Strain |  |  | | | |  | |  |
| AP4 |  | 1.00 (0.93-1.08) | | | | 0.945 | |  |
| AP3 |  | 1.02 (0.96-1.09) | | | | 0.505 | |  |
| AP2 |  | 1.04 (0.96-1.13) | | | | 0.363 | |  |
| Average |  | 1.03 (0.94-1.12) | | | | 0.501 | |  |

**Table 8. Univariate analysis of ventricular strain variables for TIA/stroke.**

|  | | | | TIA/Stroke | | | | |
| --- | --- | --- | --- | --- | --- | --- | --- | --- |
| Variables | | |  | | Univariate Odds Ratio | | P-value |  |
| Ventricular Strain |  |  | | | |  | |  |
| AP4 |  | 1.02 (0.94-1.12) | | | | 0.592 | |  |
| AP3 |  | 1.01 (0.94-1.10) | | | | 0.659 | |  |
| AP2 |  | 1.06 (0.96-1.17) | | | | 0.242 | |  |
| Average |  | 1.05 (0.95-1.16) | | | | 0.344 | |  |

**Table 9. Univariate analysis of ventricular strain variables for all-cause mortality.**

|  | | | | Mortality | | | | |
| --- | --- | --- | --- | --- | --- | --- | --- | --- |
| Variables | | |  | | Univariate Odds Ratio | | P-value |  |
| Ventricular Strain |  |  | | | |  | |  |
| AP4 |  | 1.06 (0.98-1.14)) | | | | 1.157 | |  |
| AP3 |  | 1.13 (1.04-1.22) | | | | **0.002** | |  |
| AP2 |  | 1.11 (1.03-1.21) | | | | **0.001** | |  |
| Average |  | 1.10 (1.01-1.20) | | | | **0.023** | |  |

**Supplementary Table 10. Comparative of diagnostic performance between logistic regression, multilayer perceptron and multi-task learning**

|  | Incident atrial fibrillation | | | Transient ischemic attack and stroke | | | All-cause mortality | | |
| --- | --- | --- | --- | --- | --- | --- | --- | --- | --- |
|  | Precision | Recall | F1-score | Precision | Recall | F1-score | Precision | Recall | F1-score |
| Logistic regression | 0.8591 | 0.8453 | 0.8521 | 0.8875 | 0.8864 | 0.8869 | 0.8384 | 0.7862 | 0.8115 |
| Multilayer perceptron | 0.8966 | 0.8658 | 0.8809 | 0.9002 | 0.8356 | 0.8667 | 0.9031 | 0.8722 | 0.8874 |
| Multi-task learning | 0.9440 | 0.8805 | 0.9111 | 0.9263 | 0.8819 | 0.9036 | 0.9412 | 0.9328 | 0.9370 |
